# The Neuropsychoanalytic Approach: Using Neuroscience as the Basic Science of Psychoanalysis

**DOI:** 10.3389/fpsyg.2016.01459

**Published:** 2016-10-13

**Authors:** Brian Johnson, Daniela Flores Mosri

**Affiliations:** ^1^Department of Psychiatry, State University of New York Upstate Medical UniversitySyracuse, NY, USA; ^2^Psychology, Universidad IntercontinentalMexico City, Mexico

**Keywords:** neuropsychoanalysis, neuroscience, drive, cathexis, phantoms, dynamic unconscious, pain

## Abstract

Neuroscience was the basic science behind Freud's psychoanalytic theory and technique. He worked as a neurologist for 20 years before being aware that a new approach to understand complex diseases, namely the hysterias, was needed. Solms coined the term neuropsychoanalysis to affirm that neuroscience still belongs in psychoanalysis. The neuropsychoanalytic field has continued Freud's original ideas as stated in 1895. Developments in psychoanalysis that have been created or revised by the neuropsychoanalysis movement include pain/relatedness/opioids, drive, structural model, dreams, cathexis, and dynamic unconscious. Neuroscience has contributed to the development of new psychoanalytic theory, such as Bazan's (2011) description of anxiety driven by unconscious intentions or “phantoms.” Results of adopting the “dual aspect monism” approach of idiographic psychoanalytic clinical observation combined with nomothetic investigation of related human phenomena include clarification and revision of theory, restoration of the scientific base of psychoanalysis, and improvement of clinical treatments. By imbricating psychoanalytic thinking with neuroscience, psychoanalysts are also positioned to make contributions to neuroscience research. Freud's original Project for a Scientific Psychology/Psychology for Neurologists can be carried forward in a way that moves psychoanalysis into the twenty-first century as a core contemporary science (Kandel, 1999). Neuroscience as the basic science of psychoanalysis both improves the field, and enhances its scientific and cultural status.

Psychoanalysis had its origins within neuroscience. Freud worked as a neurologist and neuroscience researcher for 20 years (Sacks, 1998) before turning his interest to a pure psychological theory, trying to explain common and previously incomprehensible disorders such as hysteria. Freud and Breuer (1893-1895) proposed using neuroscience to understand this disorder. Breuer considered the topic of energy in the nervous system while Freud described an unconscious mind. Both started to appreciate that the splitting of the psyche was not only present in hysterical patients, but in every human being. Freud wrote his last attempt to understand the mind from a neurobiological perspective in the 1895 *Project for a Scientific Psychology/Psychology for Neurologists*. Although he abandoned neuroscience in his theory, Freud thought at the end of his life (Freud, 1938) that neuroscience was still needed to support his provisional proposals of psychoanalysis.

100 years later a group of psychoanalysts and neuroscientists from all over the world renewed Freud's original approach using contemporary neuroscience resources. Solms and Turnbull (2011) have proposed a dialogue between the neurosciences and psychoanalysis that can make relevant contributions to both fields. While the field has become crowded with important research, we will summarize some of the achievements arranged thematically, and suggest that enough evidence has accreted to acknowledge that the relationship of neuroscience to psychoanalysis has become similar to the relationship of basic sciences to medicine. In fact, it was so from the beginning. New tools available in neuroscience are responsible for bringing awareness that the mind is the subject of study of many fields and perspectives and not only of psychoanalysis. Neuroscientists are building hypotheses to the study of the subjective aspects of the nervous system as simultaneously psychoanalytic clinicians are benefiting from taking the brain into account. The human being is a feeling organism that requires an integrated comprehension of both the subjective and objective. Neuroscience informs and constrains the continued building of the psychoanalytic model of the mind and its clinical applications. In the following sections, we will summarize some examples that illustrate how the inclusion of neuroscience back into psychoanalytic understanding can be of benefit for a deeper understanding of psychic determinism—a cornerstone of Freud's thinking.

## Pain, relatedness, and endogenous opioids

Pain is the most common chief complaint of medical patients (Fields and Martin, 2005). Pain Medicine has become such a complex field that it has its own fellowship program in the United States, is discussed in many pain specialty journals, and is known by physicians as a difficult symptom to address. This most common presenting complaint can become much more intelligible for both psychoanalysts and general physicians if a neuropsychoanalytic approach is used. Pain is inextricably related to the nervous system, but this subjective state that can be approached psychoanalytically.

The classical psychoanalytic approach to pain was described with beautiful insight by Thomas Szasz in the mid-twentieth century (Szasz, 1957/1975). He used the term “sensation” to describe something that is experienced equally by all, such as the glare of the sun, and “perception” as something that requires attention to bring it to full awareness, such as a “mindfulness” approach. In contrast, pain is an affect. Somatic signals are contextualized by each person's developmental history and interpersonal environment (p. 239), resulting in a partially conscious experience that a psychoanalyst can tune in to empathically. The role of the psychoanalyst is to help the patient understand their experience with more consciousness so that choices of response to pain multiply, often resulting in a more effective response to pain signals by the patient.

If a psychoanalytic approach to the pain of a patient does not take its neural substrate into account, pain can be misunderstood as a uniform experience and that all symptoms are interpretable only from the subjective point of view. Indeed defenses are arrayed unconsciously and intentionally, making the patient less anxious but also less aware of the determinants of her or his behavior. Every one of us has experienced pain. The psychoanalyst puts herself or himself in the place of the patient to help inform interpretations of unconscious determinants of behavior. In the common case of chronic back pain, patients have usually tried an array of remedies including surgeries, but the pain never stops. Consequently, many of them take opioid medication, becoming vulnerable to an addiction that involves a pathway related to subjective pain, namely the PANIC system in Panksepp's (1998) taxonomy of emotions. PANIC is activated by separation distress. The chronic back patient then comes to a psychoanalytic session to talk about a possible psychosomatic association related to their pain, but sometimes both patient and analyst are not aware of the neurochemical implications of the medication that the patient is taking. The patient will not feel in need of much human contact when taking an opioid medication, and probably will not talk much about separation anxiety, even if isolated. The transference will be modified by a physical symptom and a common medication. Understanding the interactions of pain, the PANIC system, and opioid medication in these cases is an essential clinical need. The modification of the transference by opioids, and the onset of PANIC/annihilation anxiety with the cessation of opioid use, was discussed in a psychoanalytic case report by Johnson (2010).

Another important factor can be seen in the description of fMRI results contrasting empathy with a loved one vs. a stranger. “Adopting the perspective of a loved-one increased activity in the anterior cingulate cortex and insula, whereas imagining a stranger induced a signal increase in the right temporo-parietal junction (TPJ) and superior frontal gyrus. The closer the participants' relationships were with their partner, the greater the deactivation in the right TPJ. A negative effective connectivity between the right TPJ and the insula, and a positive one with the superior frontal gyrus were found when participants imagined the perspective of a stranger” (Cheng et al., 2010; author Decety presented his work at the Athens Neuropsychoanalysis Congress in 2012). This is a fact that no psychoanalyst might get to introspectively, and some might wonder if it is useful to know at all. Apart from it showing correlates between the quality of relationships and neuroanatomical structures, which is another piece of evidence of how the mind and brain depend upon each other and are influenced by experience, it also alerts the psychoanalyst that the state of the countertransference may influence the accuracy of empathy. How close one feels to one's patient influences the brain of the psychoanalyst. The fact that a patient is taking opioid pills might affect role-responsive (Sandler, 1976) countertransference. This information may contribute to the understanding of different moments of the analyst-patient relationship that would require further investigation. Both from the clinical observation of the analyst and from neuroscientific data, this set of complementary sources of information may lead to new hypotheses on how the analytic relationship evolves during the treatment.

Pain experience may not be uniform. The discovery that multiple alleles of the SCN9A gene results in no pain, normal pain, or increased pain sensitivity (Peddareddygari et al., 2014) need to be taken into account. Due to variable genetic endowment, the analyst and the patient may have different pain systems. Neuroscience informs psychoanalysts about a potential constitutional difference. The genetic variant might be tested for if the psychoanalyst wondered about unusual pain complaints.

This information is useful to consider—that knowing someone else's experience seems to be truly impossible. However, it does not require subtracting one's empathy from a psychoanalytic encounter, or doubting its validity in all cases. That would remove a key tool from an impossible profession. The neuropsychoanalytic approach would be to use the biological information to inform clinical work, and also to imagine the possibility that the psychoanalyst's insight about developmental history and interpersonal environment might be relevant to nomothetic research. For example, the high-pain variant of the SCN9A gene was found to be present in 12% of normal controls and 40% of patients with interstitial cystitis—not 100% of interstitial cystitis patients (Reeder et al., 2013).

In summary, pain is an affect that has contributions from constitutional factors such as SCN9A gene variant, developmental history, and interpersonal environment. Empathy for the pain of a psychoanalytic patient, or the patient complaining about their bladder in a general practitioner's office, may have to do with the degree of closeness in the relationship. Genetic vulnerability is only one factor involved in a focus on the bladder. The principle of multiple function (Waelder, 2007/1936) is likely operating in a way that treaters, psychoanalytic or not, would find useful.

**Figure 1:**
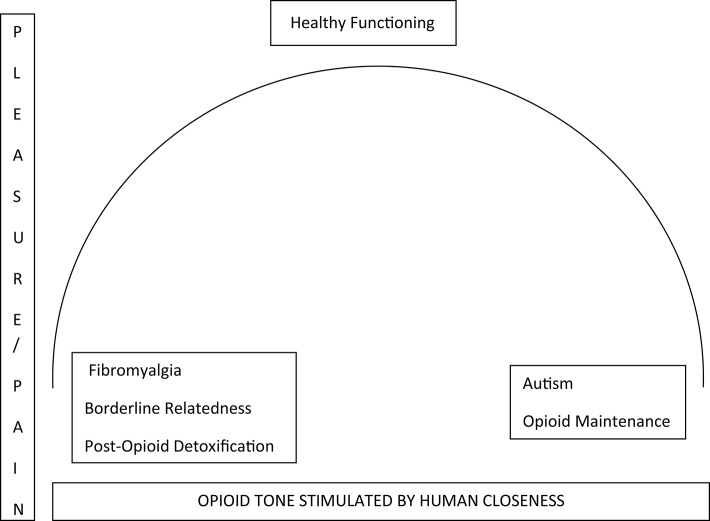
**Relationship of pleasure and opioid tone in the central nervous system subcortical pathways**.

Panksepp has shown that the pain system, an endowment of all animals to protect against tissue damage, has been adapted by social animals such as humans to modulate relatedness (Nelson and Panksepp, 1998). Being close feels good. Loss through death or separation hurts (Panksepp and Biven, 2012, p. 459). Johnson et al. (2014) elaborated some psychoanalytic consequences of Panksepp's hypotheses (Panksepp and Biven, 2012, pp. 325–328) that opioidergic activity underlies human relatedness, and that autism is a consequence of opioidergic hyperactivity (Figure 1). Johnson suggested that healthy persons use interpersonal relatedness to shift opioidergic tone in a narrow range indicated schematically by the bar at the top of the inverse U. Too much contact begins to make people uncomfortable as augmented tone becomes dysphoric. They spend time alone. Loneliness begins to make people uncomfortable as diminished tone becomes dysphoric. They initiate renewed pleasurable contact. This opioid principle can be described as a biological underpinning of Freud's pleasure principle.

Patients maintained on opioids feel and think autistically. Human contact is not needed. Their relatedness is flattened. They are emotionally unresponsive. This condition might give pause to a therapist who considers engaging an opioid-maintained patient using a treatment where relatedness is at the center of study by the analyst/analysand. For patients who dysfunctionally do little but sit on the couch and take opioid pills, a role responsive countertransference might be to feel disengaged, bored, and unable to contribute. Without the concept that opioids cause emotional withdrawal, the psychoanalyst might be misled by their lack of emotional response, with a mismatch of interpretation to the actual condition of the patient.

The concept that opioid function is contributor to relatedness has more clinical applicability. An opposite shift of opioidergic function would also have to be considered in any treatment of a patient with fibromyalgia. These patients may be emotionally unresponsive due to an autoimmune hormonal disorder that diminishes opioid tone (Ramanathan et al., 2012; Johnson et al., 2014). An approach which has promise is correcting the opioidergic deficit by pulsing the brain with a low dose of the receptor blocker naltrexone, which may provoke a rebound augmentation of function (Brown and Panksepp, 2009). Fixing the biological problem might enable the psychoanalyst to address emotional issues.

It is possible that this approach would also improve outcomes in newly detoxified patients with opioid addiction (Johnson and Faraone, 2013). Psychotherapy with interpretation used to increase human contact might also be hypothesized to augment opioidergic tone, and protect newly sober patients from persistent low opioid tone that would otherwise lead to renewed use/addiction.

A neurobiologically informed psychodynamic psychotherapist might be able to better apply transference-based techniques with patients who show compromised object relations such as borderline states by taking into account a probable chronic low opioid tone which is a product of early childhood attachment patterns (Figure 1, Johnson and Faraone, 2013). Coincidentally, these techniques might include a face-to-face format to enhance human contact/opioid tone in the transference (Figure 1, Marty, 1990) and to control regression in a closer therapeutic environment (Balint, 1968). The therapist can use body language to understand the patient's feelings more efficiently. One might observe that pain diminishes when the patient feels close/understood empathically.

Finally, what is physical pain, what is emotional pain? How are they related? By asking this question, and by not being able to give a full (saturated) explanation, a psychoanalyst is both in a position to help their individual patient think about a dysfunctional response to pain, and also to help pose questions to be investigated by neuroscience. Differentiating physical and emotional pain is complicated for fMRI research as well as psychoanalysis (Hashmi et al., 2013). A neuropsychoanalytic approach that takes both sources of information into account may be indispensable.

## Drive, instinct, and affect

A set of motivational systems is housed in the brain so that animals move through the environment to procure food, water, sex, and relationships (Johnson, 2008). Freud's concept of libido encompassed cathexis, activity, and self-preservation (Freud, 1933, pp. 95–97). Some neuroscientists (for example Pfaff et al., 2007) have proposed that Freud's libido can be related to generalized brain arousal systems. However, many contemporary psychoanalytic theories reject the concept of drive (Eagle, 2011, p. 252). Neuropsychoanalysis has embraced Panksepp's formulation of seven basic instinctual systems and has used his proposal to discuss the concepts of instinct, drive, and affect.

Panksepp mapped these behavioral systems by systematically stimulating brain areas electrically or chemically, and then observing the resulting animal behaviors (Panksepp and Biven, 2012, p. 25). He has turned the usual caution of animal researchers against “anthropomorphism” on its head, suggesting that all animals have evolved neural systems for survival and for success in navigating peer social environments, and that they are felt. He is known for the discovery, by empathically observing rat behavior and then by use of a transducer for high-pitched sounds, of rat laughter (Panksepp, 2007).

The seven basic emotion systems that Panksepp has described are shared by all mammalian brains. Emotional systems are subcortical. They are the SEEKING, LUST, CARE, PLAY, PANIC, RAGE, and FEAR systems. Panksepp uses capital letters to identify a concrete neural circuit, as opposed to the abstract use of these same words.

The SEEKING system is superordinate, and has been considered by some neuropsychoanalysts (for example Yu, 2001b) an analog of Freud's libido. Drive may be considered the psychological manifestation of SEEKING, an urge to do work in order to obtain a desired goal. Panksepp's aphorism is that SEEKING is “a goad without a goal.” SEEKING energizes organisms to explore their environment. It produces for the animal an exciting, optimistic and engaging affect. It reaches out to the other six basic systems to turn them on when needed or the other way around; other instinctual systems tell SEEKING what to search for (Panksepp and Biven, 2012); giving the goad a goal.

SEEKING can search for basic items to satisfy needs in the environment, such as food, water, sex, relationships, and if addicted, drugs (Johnson, 2008). Some of these items may not involve pleasure because SEEKING as all other basic emotion systems learns and is conditioned by life experiences. If one grows up in a context of basic trust (Erikson, 1950) from empathic and caring parents, relationships may be sought in adulthood with a SEEKING system that has been tuned by learning to look for affective quality in relationships with others. But if parents were abusive or neglecting, food, love, and an occasional unexpected smack may make the bond with that parent more intense. Pathological relationships may be sought because the SEEKING system has been organized by learning to look for unexpected attacks related to the lack of basic trust. We urgently pursue our goals whether pleasant or not. The SEEKING system should not be equated with a reward or pleasure system. SEEKING is not the only rewarding feeling in the nervous system and it is not quite rewarding in itself; its activation results in anticipated excitement. If no satisfaction comes, it shuts down. The feeling is related to frustration.

SEEKING's main neurotransmitter is dopamine. Pleasure is a separate system, with mu opioids as the most important contributor (Robinson and Berridge, 1993; Panksepp, 1998). What we want, and what we like, belong to two different brain systems. The pleasure system will only be activated when an object that satisfies a need is found. SEEKING looks for the object needed. The pleasure system enjoys the interaction with the satisfying object. Dopamine drives motivation while mu opioids give the feeling of pleasure.

Other instinctual systems are activated according to setting. Animals move to the side of the cage where electrical stimulation turns on these good feelings (Panksepp and Biven, 2012). They move away when the bad feelings are turned on. We all feel “good” when we are in the PLAY, CARE, LUST mode. We all feel “bad” when we are in the FEAR, RAGE, PANIC mode.

PLAY, CARE, and LUST are positive, socially engaging systems and are thus important to understand two-person psychology or even the analytic third concept proposed by Ogden (1994). PLAY is built into all mammals so that we rehearse social roles and conflicts without risking consequences. PLAY for children requires a rough and tumble interaction format that elicits an affect of social joy. Wright and Panksepp (2012) advises that psychotherapy be carried out in the PLAY mode. Observing patients who are not capable of PLAY in psychotherapy gives important information regarding (psychoanalytically) genetic experience and current interpersonal difficulties. Good and fun experiences with peers are pleasurable and will be repeated reinforcing the experience of being in company as an important agenda to promote survival. LUST is the system that makes mammals look for sexual partners, not only looking for pleasure, but also for the survival of the species. CARE is a system activated whenever someone perceives another being in need of help. It is the basis of maternal love and it may have a lot to do with the psychotherapeutic feeling involved in helping patients at certain stages of psychoanalytic treatments, particularly with pre-Oedipal patients (Balint, 1968; Marty, 1990). The CARE system is vital to the understanding of the relationships between mothers and their offspring so relevant to psychoanalytic developmental theory. The activation of a mother's CARE system makes her full with love that will contribute to her protecting a fragile baby that could not survive without her CARE.

FEAR, RAGE, and PANIC/GRIEF have as their basic function tissue protection. As opposed to the pleasant feelings generated by SEEKING, PLAY, CARE, and LUST, these systems generate dysphoric affect. FEAR is the feeling we all get when exposed to dangerous situations that compromise survival. It is not interpersonal. It provides the animal with various choices when endangered. RAGE exists to deter attackers when flight is impossible. Panksepp points out that the “fight/flight” term describes engagement of RAGE or FEAR (Panksepp and Biven, 2012, p. 200), and actually refers to two systems, not one.

PANIC/GRIEF underlies the need to attach to others for survival. It is the perfect complement of the CARE system as seen in mother-child interactions. Activation of the PANIC system is seen when animals separated from their parents call out with “distress vocalizations” to help the protective locate the child. If an animal is separated from its caring object for too long the PANIC separation distress vocalizations cease. Watt and Panksepp (2009) suggest that the original function of vocalization shutdown was to protect animals from signals that might make them vulnerable to attack from predators. Unrelieved alarm at separation finally terminates by entering a shutdown mode, a state of waiting to be found can be understood as a freezing reaction, or—depression. This model corresponds with the literature on psychotherapy and antidepressant medications for depression. Mild major depression responds about equally well to psychotherapy or medication, but more severe and chronic depression requires antidepressants (de Matt et al., 2007). As Watt and Panksepp would say (Watt and Panksepp, 2009), antidepressants change the brain, allowing the patient to return to the broad plateau of anxiety where interpersonal difficulties in being close can be resolved with the psychoanalyst. Panksepp (Wright and Panksepp, 2012) has suggested that treatment must activate the SEEKING and PLAY systems as well as deactivate the PANIC/GRIEF system to treat depression.

An example of dysregulation of the basic emotion systems can be seen in the adoption of psychotoxic drug use. The person feels constantly bad even when the traumatic experiences that caused the negative affects are diminished or blocked by defenses. The person tires of the unpleasant feeling and wants relief. Experimenting with psychotoxic drugs the person discovers the direct neurochemical impact on her or his subjectivity. The patient has become a “psychiatrist” herself or himself, understanding that there is a neuropsychodynamic relationship between mind and brain. Taking the psychotoxic drug creates a “high.” For example, a patient who had been terrorized by a sadistic father, and who met the diagnostic criteria for posttraumatic stress disorder, described the “high” of alcoholic drinking as the feeling of relief from constant reactivation of her terror by random stimuli in her environment.

The basic emotion systems constitute knowledge about subjective states that are always conscious because affect is always conscious. Taking it into account in psychoanalytic theory propitiates metapsychological conceptualizations such as the conscious id proposed by Solms and Panksepp (2012) and Solms (2013). It also facilitates clinical work, particularly with pre-Oedipal patients who do not respond well to the classical psychoanalytic technique, but who require modified approaches mainly based on empathy (Balint, 1968).

If we want something, or feel a certain way, the primary feeling comes from brainstem areas. Consciousness is obliterated by brainstem lesions, and preserved in cortical lesions (Solms, 2013). Solms proposed that Freud's id, the origin of drive, is represented in the brain by consciousness that cannot be without affective experience—the primary feelings being related to Panksepp's instinctual systems with secondary and tertiary elaboration into more complex emotional experiences such as love, hate, disgust, appreciation, gratitude, etc. For Solms and Panksepp (2012) if affect is always conscious, then the id is conscious. The ego, first a body ego, starts with exteroceptive experiences that register cortically. Thinking can be repressed, but emotions cannot. The psychoanalyst treating a patient may notice that they are taken over by feelings most certainly provoked in part by their experience, but not be consciously aware of why they may feel bored, angry, sexual, afraid, or depressed. The lack of conscious connection is a function of inhibition, probably cortical/ego inhibition.

Object representations are cortical. They stabilize and facilitate experiences in the world. Knowing that a certain person always acts the same makes contact less effortful than on first meeting them. Solms (2013) suggested that working memory underlies object representations and that planning ahead is required to inform subsequent encounters so that the interaction occurs with optimal relatedness. This mechanism underlies Freud's concept of bound energy. If life is always completely predictable, one is living in Freud's Nirvana principle. Thinking is non-conscious and not effortful. One always expects what one reality provides.

Free energy has to do with SEEKING as it presses for activity. One is unhappy and unfulfilled when one needs something, even though it may not be clear exactly what that might be. This is the energy that reaches quiescence with gratification. The SEEKING system knows what to look for only when given feedback from other basic emotional systems. What we SEEK does not always lead to optimal results. There may be learning experiences that lead to solutions that make us approach harmful stimuli such as the cases in which a narcissistic injury can contribute to pathological object relations in adulthood. More sophisticated tertiary process thinking/conscious awareness and reflection about what we feel and think, possibly facilitated by analysis of a transference relationship, may allow shifts in goals and behaviors. The inhibitory centers of the prefrontal cortex are related to ego and superego functions. Consciousness seems to enhance survival because we can reflect and think about the very fact of being alive (Solms, 2013).

## Dreams

Psychoanalysis was officially born in 1900 with Freud's dream theory. The mind was primarily unconscious. Dreams became the royal road to the unconscious. Thus, dreams had to be interpreted. This theory was predominant until psychoanalysis suffered a cultural credibility defeat with the publication of the activation-synthesis model by (Hobson and McCarley 1977). They claimed, with neuroscience evidence, that dreams were the meaningless froth of random pontine firing during rapid eye movement (REM) sleep (Solms, 1997). REM sleep was eliminated by transection of cat brains at the level of the pons. Dream interpretation was therefore the awakened person's best attempt to put some meaning to a pontine message that never had any meaning. The cultural narrative became that psychoanalysts were collecting fees from patients for fraudulently abetting this bizarre, empty meaning-finding activity.

Cats whose brains have been transected at the pons lose their rapid eye movements. But they cannot tell about their dreams. The claim that dreaming depends on REM sleep and that dreams are meaningless pontine signals has been singlehandedly reversed by Solms' painstaking work with 361 subjects who were asked if they noticed a change in their dreaming since the onset of a known neurological illness (Solms, 1997, p. 83). His analysis of the results of this case series led him to a hypothesis that the dream pathway originates in the ventral tegmental area (VTA) of the midbrain, runs through the nucleus accumbens (NA) via basal forebrain white matter tracts and then through the mesocortical and mesolimbic dopaminergic systems. Hobson and McCarley's results could then be understood as pontine-geniculate-occipital spikes of acetylcholine-fueled neural activity further energizing the basal dopaminergic thinking involved in non-REM (nREM) dreaming when these cholinergic transmissions were turned on as part of the transition from nREM to REM sleep. Solms concluded that VTA/NA activation stimulating more rostral structures produces dreams. According to interviews of patients with lesions, dreaming ceases when basal forebrain white matter and/or the temporo-parietal-occipital junction are damaged as opposed to pontine lesions, which do not cause dream cessation. Dreaming is dissociated from REM sleep. It can occur during NREM and does not depend on pontine mechanisms.

A further consilience is that Solms' dreaming pathway is exactly Panksepp's SEEKING pathway. Dreaming and SEEKING involving the same neural pathway is congruent with Freud's (1900) hypothesis that every dream originates with a wish (Solms, 2000), a motivation. Therefore, dreams are turned on by items that are urgently wanted. Complications of obtaining these entities are further manipulated via dreamwork to anticipate conflicts and barriers in the social environment of the dreamer. Freud's work with dreams, and that of every psychoanalyst since, has been vindicated by the work of Solms.

This work has been further developed by Yu (2001a,b), whose independent analysis confirmed both the neural pathways involved in dreaming and suggested (Yu, 2001b) the congruence of Freud's (1923) “great reservoir of libido” and Panksepp's SEEKING system. In another vindication of Freud's work, Colace (2012) has shown a change in the dreams of children at about 5 years of age, corresponding with the onset of superego function. Dreams of younger children are simple and directly anticipatory of gratification. Dreams after the age of five have a tortured, complex nature, as if there are internal barriers to gratification. A main point of Colace's (2012) is that with a thorough command of Freud's disguise-censorship model, nomothetic studies that he has conducted provide strong evidence that internal conflict between wish and moral consciousness appear in dreams.

Levin (Levin et al., 2009) has also considered the neuroscientific data of importance to dream work, adding that the involvement of the SEEKING system also refers to pending topics in the life of the dreamer. Levin considers the motivation of SEEKING to play an important role in problem resolution. This is another contribution to the wish fulfillment of dreams, namely that of exercising possible answers or strategies for current problems in a person's life.

Finally, Solms (2000) suggested that the same neural system that involves dreaming also involves drug seeking. This idea was further developed by Johnson (2001, 2003) and Colace (2004, 2014) (Colace et al., 2010) as the neural basis of drug dreams. Johnson (2003, 2011) hypothesized that dreams formed a neural readout of brain changes that represent a shift from a psychological form of addiction as a solution to emotional problems to the brain-based illness of compulsive drug use. Only patients whose brains have been exposed to cocaine or heroin have dreams that involve looking for and trying to use these drugs. Dreams that involve SEEKING alcohol probably signal the transition from alcohol abuse (psychological addiction) to alcohol dependence (physical addiction; Johnson, 2011).

## Cathexis

Although Panksepp (1999, pp. 28–29) and Yovell (2008) are reluctant to claim any 1:1 mapping of Panksepp's instinctual systems onto Freud's libido, Johnson (2008) has found another consilience by using the SEEKING system to explain the Freudian concept of cathexis. This psychoanalytic concept is on the endangered theory list. The assertion that humans are object-seeking by nature, and that this has nothing to do with “drive,” has been widely adopted as a bedrock of psychoanalysis (Eagle, 2011, p. 54). Panksepp's elaboration of instinctual systems that are activated by SEEKING helps resolve this dilemma within psychoanalysis. The PANIC, RAGE, CARE, LUST, and PLAY systems are object-related. Mammalian brains are pre-wired to bond to a care-giver for survival. CARE and LUST are the basis of love (Panksepp and Biven, 2012, p. 38). PLAY demands involvement with peers for social joy. These neural systems give a goal to the goad of the objectless SEEKING system, telling it what it is looking for. PANIC warns organisms not to avoid relationships by giving a dysphoric signal when closeness is disrupted. RAGE provides an array of possible responses to uncomfortable impingements from others. SEEKING does not act randomly but is given information by other basic emotion systems to know what to seek for. CARE, LUST, and PLAY systems need the SEEKING to successfully cathect objects.

Johnson (2008) described cathexis as the consequence of repeated stimulation of the SEEKING system during early development. Animal research studies demonstrate that inhibition of dopamine, mu opioid receptors, and hormones such as oxytocin and prolactin, each inhibit the formation of partner preference or maternal-infant bonding. Knocking out any one of these systems results in behaviors that do not persist, such as mating without the formation of partner preference. Bonding is produced by dopaminergic drive coupled by hormones to interactional experiences—in prairie voles and rats (Insel, 2003). These concepts can be extended to humans.

Johnson paired neurobiological information with Freud's (1920) paper, “Beyond the pleasure principle,” to give further evidence for Freud's hypothesis that drive is necessary to form attachments. Using the neuropsychoanalytic principle of dual aspect monism the paper demonstrated that animal research is congruent with Freud's concept of a conflict between the pleasure principle and a force that is, “More primitive, more elementary, more instinctual than the pleasure principle which it overrides” (Freud, 1920, p. 23). If one is SEEKING relationships based on difficult experiences developed in childhood that do not function adaptively in one's adult life, repeated unpleasant experiences result. The cathexis system had been tuned by drive-related learning to look for those affective needs that were not satisfied in the past. Unsatisfied affective needs survive despite age. The very process of development is compromised frequently resulting in pre-Oedipal pathologies. Traumatic emotions do not expire and so they will try to cathect current objects. In these cases a drive/pleasure conflict can be made conscious with psychoanalytic treatment, resulting in more adaptive relationships.

Johnson was careful to state that the existence of drive in no way negates the current focus on how urgently humans want to make relationships. It gives a neural basis for this urgency. By using the clear difference between neural systems that are involved in wanting and liking (Robinson and Berridge, 1993, 2000; Panksepp, 1998), the psychoanalyst can use of neuroscience to extend our definition of “neurotic.” Patients with a series of complaints about how their lives are not working out may be trapped by conflicts between these neural systems. They may be urgently pursuing goals with their SEEKING system that do not line up well with pleasure. What they want is not what they like, resulting in neurotic misery. The person adopts inflexible patterns to try to adapt, which ignore the signals that affective consciousness constantly provides.

According to Watt and Panksepp (2009) depression is the result of a dysregulation mainly of the opioid PANIC system, which in turn leads to the shutdown of the SEEKING system. This combination of high PANIC and low SEEKING seems to lead to a hopeless feeling. The resulting exhaustion can lead to the wish to die in severely depressed patients, the product of endlessly SEEKING relationships which are unpleasant and destructive. A critical period is present in the evolution of the SEEKING system in early life. If relationships have been affectively confusing or traumatic, such dysregulation is repeatedly sought, leading to essential (Marty, 1990), or anaclitic depressions (Bergeret, 1974, 1975).

Making a relationship with patrons through activation of the cathexis system during drug sales, and thereby fulfilling a drive that was created by the neural changes induced by the drug, entrepreneurs of the addictive drug industry make close relationships with customers who often die from using their product. The compulsive use of addictive drugs is based on cathexis to the seller of the drug as well as to the drug itself (Johnson, 2008, 2009, 2013). The compulsive use of drugs is based on a dysregulation of the SEEKING system that recruits cues to build conditioned stimuli and responses. Affective misery is shut off by drugs that quiet experiences of PANIC, FEAR, and RAGE. This way of thinking about compulsive behaviors with drugs or food gives the psychoanalyst a skill set that is essential to help patients who suffer from behaviors that kill. Alcohol causes 4% of deaths worldwide (World Health Organization, 2010). Cigarettes kill about four million persons per year (World Health Organization, 2008). Love, cathexis, and death are linked in this way. Death from cigarettes is not due to a direct wish to die, otherwise smokers would simply kill themselves in a faster way. Smoking is the result of complicated and differentiated motives (Wurmser, 1974). Nicotine dysregulates several neural systems, including SEEKING—which constantly demands nicotine. Addiction is one of the illnesses that require understanding both subjective experience and drug-induced brain changes.

## Dynamic unconscious

Non-dynamic psychology has provided a way of explaining that thought is either implicit/procedural or explicit/conscious (Rosenblatt, 2004). For example, the first day you drove to your new job, you explicitly thought about each turn. Everything you did was conscious.

By now your car procedurally drives itself to work while you listen to the radio or talk to a passenger. If you run into new construction you shift to explicit thinking so that you still get to work. You turn off the radio or stop talking to your friend, and pay attention to the way around the construction. This process does not involve repression or dissociation. No defenses are employed. There is nothing “dynamic” about procedural unconscious behaviors such as driving one's car. Your behavior is descriptively but not dynamically unconscious. Neuroscience has referred to procedural, automatic behaviors as non-conscious. They economize the processes of the nervous system once learned.

On the other hand, you may “forget” to attend a meeting that you really didn't want to go to, but consciously would have made yourself go to if it weren't for the handy defense of repression. You “remember” the meeting as soon as it is too late to go. Then you feel bad. The difference between thinking that is not conscious but not made unconscious (descriptively unconscious) and material that is barred from conscious thought (dynamically unconscious) is seen in these examples. Current psychoanalytic thinkers favor concepts such as “unformulated experience,” “implicit relational knowing,” and “habitual relationship patterns” (Eagle, 2011, pp. 107–131), while the old Freudian formulation of a stimulus barrier that protects the ego from unacceptable information is going the way of drives, dreams, and cathexes.

As with dream theory, Solms, with Kaplan-Solms, had the original insights about using neurologically impaired patients (Kaplan-Solms and Solms, 2000) to demonstrate striking evidence for the psychoanalytic concept of mechanisms of defense. One example is the anosognosia of right hemisphere lesion patients. Non-analytic neuropsychologists and neurologists invoked concrete concepts based on their guesses about right hemisphere function. Maybe the problem had to do with inattention, perhaps with the right hemisphere being responsible for conceptualizing negative emotions, or for monitoring the body (Solms and Turnbull, 2002, pp. 263–264).

The neurologist can benefit from a psychoanalytic explanation to understand why the patient denies that their whole left side is paralyzed. Psychoanalysts can see the evidence of the unconscious laid out before them in a way that is stark and well-delineated. Taking out a key right parietal part of the brain does not make the person unable to function emotionally. It eliminates the right parietal sense of location. Kaplan-Solms and Solms' (2000, pp. 172–199) formulation regarding conscious indifference about the paralyzed left side was explained using Freud's (1917) concepts from “Mourning and Melancholia.” In a left parietal stroke, position sense is preserved. The body is an object. The patient mourns the loss of movement of the right side of the body. Object love allows normal grief.

In a right parietal stroke, the patient is unable to understand where the paralyzed left side lies. Some right parietal stroke patients deny their paralysis and act as though nothing were wrong with them. Kaplan-Solms and Solms interpreted this feature as a narcissistic defense by which the patients deny a painful aspect of reality. Unconsciously they know about their paralysis, but their body location is impaired. An idealized “good body” is introjected, protecting the stroke patient from the horror of their loss. Unfortunately, a “bad body” is also introjected, and is hated. The hatred can be experienced toward the self, resulting in a melancholic depression, or projected into others, resulting in an experience of being loathed by others. These regressive dynamics are initiated by the loss of the sense of location, of reality, that had been produced by the loss of location sense that was a function of the destroyed right parietal cortex. In neurologically impaired patients, evidence of dynamically unconscious thinking, and the defenses that keep material unconscious, can be easier to observe and describe using the clinico-anatomical method.

Another example of the dynamic unconscious functioning can be found in Bazan's (2011) innovative formulation of “phantoms.” Whether you get to work with explicit or implicit thinking, there is no discomfort as long as you get there. The brain has two feedback systems to monitor movements. It makes a calculation from the somatosensory cortex about where the car was estimated to go. The proprioceptive system—vision and muscle receptors—says the car has gone where the brain willed it, the “efference copy.” The two sensory systems line up. You feel good when you get where you are going.

Skipping the meeting when you had “intended” to go, results in discomfort based on a lack of congruence between intention and behavior. A “phantom” intention has no feedback from proprioceptive system that you were at the meeting. Defenses undo intentions by making them unconscious and therefore unable to be acted on. The residua produce a feeling of discomfort.

Bazan linked this set of ideas with word representations that are seen as vulnerable to repression and substitution. She suggested that the starting point of unconscious thinking is drive. The drive comes into conflict with the social milieu of the person. The drive itself cannot be repressed, only the corresponding motor execution. For linguistic intentions, the phantoms are phonemic. She uses the compulsions of the Rat Man (Freud, 1909) to show displaced action based on word representations, shifting from an intention to marry (in German, “heiraten”) his beloved to various substituted rats: Frau Hofrat—the governess he had sexual play with as a child, fear of rats, the German term “Raten” which refers to payments to be made. Words or ideas that are repressed cause the phantoms to be undischarged, creating anxiety.

A constant underlying dynamic of compulsion is undoing of unconscious intentions. This was Dodes' (1996) explanation of addiction as a kind of compulsion where the aggressive intention to harm another is displaced into an addictive behavior rather than a compulsive undoing of the intention. The sober person who does not have good recovery (a dry drunk) is filled with anxiety about aggressive urges which have not been carried out. The minute the person decides to drink (Dodes, 2002, p. 4) they feel better. Bazan might say that they know their aggressive urges will be fulfilled. They know the aggressive phantoms will be carried out via displacement; the aggressive act is not done consciously, but rather is expressed as an addictive behavior.

These phantoms are based on a melancholic state (Freud, 1917) in which past narcissistic injuries related to the primary object lead to a RAGE activation where the introjected object needs to be destroyed due to its negative split valence quality. The compulsive behavior is related to the memory of pain because of how the object failed to give narcissistic nurture at a critical time in early life. This formulation reframes and further explains Dodes' (1996) assertion that addictive behaviors are both directed toward current objects and are also driven by childhood memories of traumatic helplessness.

## Is the practice of psychoanalysis like the practice of medicine?

Harrison's Textbook of Medicine (Kasper et al., 2005, p. 1) began with a chapter, “What is expected of the physician.” Their assertion was, “The accelerating pace of changes in medicine stems from an explosion of scientific information and the need to blend this information into the art and practice of medicine.” Psychoanalysis originated in neuroscience precisely attempting to understand complex symptoms such as the ones observed in hysteria and that traditional medical approaches had failed to treat. Freud used all his neurological background to develop the psychoanalytic proposal to focus on what medicine had not considered: the mind. The mind was not seen as an isolated entity, but was related to the body and the nervous system. Metapsychology is based on neuroscience and is thus the original basis of clinical psychoanalytic practice. Current knowledge from neuroscience can offer new perspectives from which clinical phenomena in psychoanalytic practice can be better understood.

Neuropsychoanalysis is a twenty-first century development that has at its core the concept of dual aspect monism (Solms and Turnbull, 2002). Whether phenomena are evaluated empathically, or through measurements and statistics, it introduces an artifact of perception. Empirical data is filtered through the means by which it is made. Therefore, we are in the delightful position of being able to make observations by psychoanalytic clinical means that can also, perhaps with some technical difficulties, be made with genetic testing, animal observation of homologous behaviors, fMRI scanning, or some other nomothetic approach. The same event can be observed in stereo. Each approach informs the other. As Turnbull et al. (2004) put it, “Neuropsychoanalysis (carries) a useful balance between two quite distinct approaches: a clinically based tradition that has a long history of generating provocative ideas, and an experimentally based tradition that can test these ideas in a more rigorous fashion.”

The discussions above reflect this approach. Nothing is lost from psychoanalysis. Psychoanalysis can only benefit from an open dialogue with its old basic science, neuroscience, but through a twenty-first century trip back to its origins. More psychoanalytic insights and new theories are likely to appear when psychoanalysts make observations tuned by neuroscience information, such as Bazan's concept that anxiety is driven by repressed, urges, “phantoms,” or the concept that physical pain and human relationships may be modulated by the same system. The brain is the organ of affects and subjectivity. It is the organ of the mind.

Psychoanalysis is a theory but also a technique in practice that then benefits directly from information coming from other fields, particularly neuroscience, the study of the nervous system which increasingly includes subjective aspects. Neuroscience was and can again become the basic science of psychoanalysis. Famous cases such as Phineas Gage and HM have shown the impact of a mind-brain relationship. Psychosomatics has also given a connection between stress and body reactions involved in various diseases because of autonomic dysregulation (Alexander, 1950; Marty, 1990). Neurochemical manipulations by psychiatrists or addicted persons have demonstrated an impact on subjective feelings. Studies have shown modifications that psychotherapies have on the brain (Buchheim et al., 2012, 2013; Bastos et al., 2013; Abbass et al., 2014; Fournier and Price, 2014). It may be necessary to learn about the mind-brain relationship if one practices any sort of psychoanalytic treatment.

Fotopoulou et al. (2012) explained that, “Neuroscience…shares an important goal with Freudian metapsychology, namely to generate an accurate, large-scale model of the mind…” She described psychodynamic neuroscience as:

1. forming and testing hypotheses that derive from a wider theory of the mind, and re-integrating findings within the same theory;
2. forming and testing hypotheses that have been informed by years of clinical practice, and thus indirectly taking subjectivity into account;
3. ascribing to mental processes an ontological status that is as real as that of neural processes, and hence capable of causally influencing the latter;
4. firmly acknowledging the epistemological limitations of the discipline as a third-person, neuroscientific endeavor.

“I anticipate this focus will allow greater progress in understanding the neurobiological basis of the mind, as well as avoiding the extreme materialism and reductionism of some other neuroscientific approaches.”

Challenges to neuropsychoanalysis from outside of psychoanalysis have to do with reestablishing the value that psychoanalysis used to have for science. Psychoanalysis has gone from the dominant paradigm in psychiatry in the 1960s to a marginalized field. Multiple investigators report that applications for research funding are dismissed as coming from a discredited source. Solms and Turnbull (2002, p. 299) wrote, “Both of us have been in professional situations—more often than we like to remember—where our interest in psychoanalysis has made it difficult to maintain the respect of our colleagues, the esteem of our students, and the willingness of journal editors to publish our work.”

Neuroscience papers use behaviorist language to explain behavior that specifically leaves out the brain. For example, Volkow and Baler (2014) describe the “reward system” of the brain rather than discussing drives or SEEKING. As explained by Panksepp (1998, p. 12) behaviorist psychology regarded the brain as a black box. The “science” of psychology was to impinge on animals and count resulting behaviors. This behaviorist approach to neuroscience, using concepts that have nothing to do with the brain, impoverishes. Animal behavior is deeply comprehended when one understands that animal actions are led by their affects. Human beings are no exception to the fact that animals are profoundly emotional. Emotions are based in neural circuits that provide our subjective states of the mind. Neuropsychoanalysis offers an approach to brain-based psychological concepts that can enrich both neuroscience and psychoanalytic understanding.

In Govrin's (2006) critique of postmodern relational psychoanalysis he avers that one of the most important contributions of positivist psychoanalysis is the ability to build “thick” descriptions of phenomena. To build up the material basis of psychoanalysis keeps it from drifting into idealism (Johnson, 2006). Kandel (1999) in his famous paper about uniting biology and psychology spoke of psychoanalysis as being biology's best psychological partner. His criticism highlighted topics that need thorough reconceptualization and discussion to help psychoanalysis to be even better, rather than be somehow destroyed or discarded by a neuroscience perspective. If psychoanalysis can dialogue its concepts and findings with other fields of knowledge, then it is mature enough to keep making progress toward a better understanding of the mind. Dual aspect monism allows a complexity of conceptualization that is missing from postmodernist cautions about inability to know what is really true (Govrin, 2006).

## Conclusion

Neuropsychoanalysis continues Freud's original endeavor of “psychology for neurologists.” It offers a way forward that preserves and extends the empathic insights of psychoanalysts by affording a second way to “know.” It allows the building of more complex concepts without eschewing the postmodern skepticism of relational psychoanalysis. In fact, skepticism is directly built into the scientific method.

The ideographic ideas of psychoanalysis have nothing to fear from the nomothetic procedures of neuroscience. An example given above was Hobson and McCarley's claim that dreams were nothing but random pontine signals. They tried to discredit psychoanalysts as if psychoanalysts had been collecting fees and spending time explaining neural noise that had no meaning. Sophisticated neuropsychoanalytic work by Solms and others has put psychoanalytic dream work on solid ground. As Solms (2013) stated, “Neuroscience is no more the final court of appeal for psychoanalysis than psychoanalysis is for neuroscience. The final court of appeal for psychoanalysis is the *clinical* situation.”

We anticipate that some psychoanalytic ideas will need revision and reconsideration when considered against the underlying neural systems involved in creating our psychology. This ability only gives psychoanalysis more credibility for the theories that have clear neuroscience evidence such as drive, including a drive to be related, the structural model, the value of dreams, cathexis, and the validity of the concept of the dynamic unconscious. Neuroscience is naturally the basic science of psychoanalysis. In the twenty-first century these two fields can only benefit from working together again after years of dissociation. An integrative perspective can create dialogues leading to better conceptualizations and techniques to be applied in the clinical situation. The human mind longs for a better understanding about itself. Neuropsychoanalysis is the way. The precept that neuroscience is the basic science of psychoanalysis is of value to the field.

## Author contributions

BJ and DF collaborated for several years to produce this paper. BJ wrote the first draft. Each has taken turns writing and rewriting.

### Conflict of interest statement

The authors declare that the research was conducted in the absence of any commercial or financial relationships that could be construed as a potential conflict of interest.
